# i4mC-Mouse: Improved identification of DNA N4-methylcytosine sites in the mouse genome using multiple encoding schemes

**DOI:** 10.1016/j.csbj.2020.04.001

**Published:** 2020-04-08

**Authors:** Md. Mehedi Hasan, Balachandran Manavalan, Watshara Shoombuatong, Mst. Shamima Khatun, Hiroyuki Kurata

**Affiliations:** a Department of Bioscience and Bioinformatics, Kyushu Institute of Technology, 680-4 Kawazu, Iizuka, Fukuoka 820-8502, Japan; b Japan Society for the Promotion of Science, 5-3-1 Kojimachi, Chiyoda-ku, Tokyo 102-0083, Japan; c Department of Physiology, Ajou University School of Medicine, Suwon 443380, Republic of Korea; d Center of Data Mining and Biomedical Informatics, Faculty of Medical Technology, Mahidol University, Bangkok 10700, Thailand; e Biomedical Informatics R&D Center, Kyushu Institute of Technology, 680-4 Kawazu, Iizuka, Fukuoka 820-8502, Japan

**Keywords:** Mouse genome, Sequence analysis, Sequence encoding, Machine learning

## Abstract

*N*4-methylcytosine (4mC) is one of the most important DNA modifications and involved in regulating cell differentiations and gene expressions. The accurate identification of 4mC sites is necessary to understand various biological functions. In this work, we developed a new computational predictor called i4mC-Mouse to identify 4mC sites in the mouse genome. Herein, six encoding schemes of k-space nucleotide composition (KSNC), k-mer nucleotide composition (Kmer), mono nucleotide binary encoding (MBE), dinucleotide binary encoding, electron–ion interaction pseudo potentials (EIIP) and dinucleotide physicochemical composition were explored that cover different characteristics of DNA sequence information. Subsequently, we built six RF-based encoding models and then linearly combined their probability scores to construct the final predictor. Among the six RF-based models, the Kmer, KSNC, MBE, and EIIP encodings are sufficient, which contributed to 10%, 45%, 25%, and 20% of the prediction performance, respectively. On the independent test the i4mC-Mouse predicted the 4mC sites with accuracy and MCC of 0.816 and 0.633, respectively, which were approximately 2.5% and 5% higher than those of the existing method (4mCpred-EL). For experimental biologists, a freely available web application was implemented at http://kurata14.bio.kyutech.ac.jp/i4mC-Mouse/.

## Graphical abstract

In this work, we developed a new computational predictor called i4mC-Mouse for identifying 4mC sites in the mouse genome. A computational framework of i4mC-Mouse.

**Figure:**
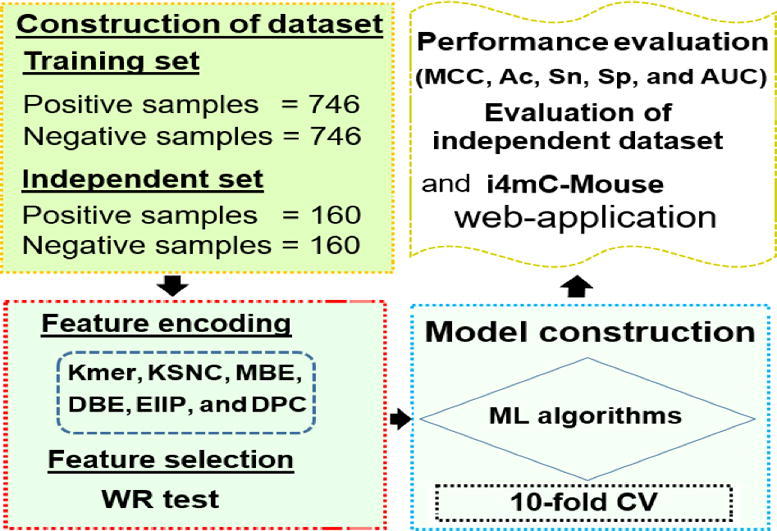

## 1 Introduction

In both prokaryotes and eukaryotes, N4-methylcytosine (4mC), 5-Methylcytosine (5mC), and N6-methyladenine (6 mA) alterations can regulate various functions including genomic imprinting, cell developmental, and gene expressions, and play crucial roles in the genomic diversity [1], [2]. The 5mC modification is a common type of methylation alteration and well-explored that exemplifies an important role in biological developments [3], [4] that are associated by the various diseases such as diabetes, neurological, and cancer [5], [6]. The 4mC modification is also an effective methylation that defends the self-DNA from being degraded by restriction enzymes.

Until now, many experimental methodologies, such as mass spectrometry, methylation-precise PCR, and Single Molecule of Real-Time (SMRT) sequencing [7], [8], [9], [10], have been efficiently used to identify the epigenetic 4mC sites. The exact dataset of modifications of 4mC sites is still limited due to the shortage of experimental identification approaches. Moreover, the aforementioned experimental approaches are labor-intensive and expensive works. Thus, computational tools are required for analysis of the accessible big data on the genome of mouse so as to allow the identification of novel 4mC sites, while shedding light on their mechanism [11], [12]. Several computational approaches have been proposed by using the recently constructed database named MethSMRT [13] to predict 4mC sites from seven different species, i.e. *E. coli*, *G. subterraneus*, *A. thaliana*, *D. melanogaster*, *C. elegans*, *G. pickeringii,* and *Rosaceae genome.* [11], [12], [14], [15], [16]. To the best of author's knowledge, only one predictor is available for the 4mC sites in the mouse genome, named 4mCpred-EL [11]. This method implemented multiple encodings and machine learning (ML) algorithms, which was applied to the dataset derived from the MethSMRT. Although the 4mCpred-EL yielded encouraging results, there is still room for further enhancement, probably because the employed feature information is not sufficient to capture the discriminative information between the two classes.

**Fig. 1:**
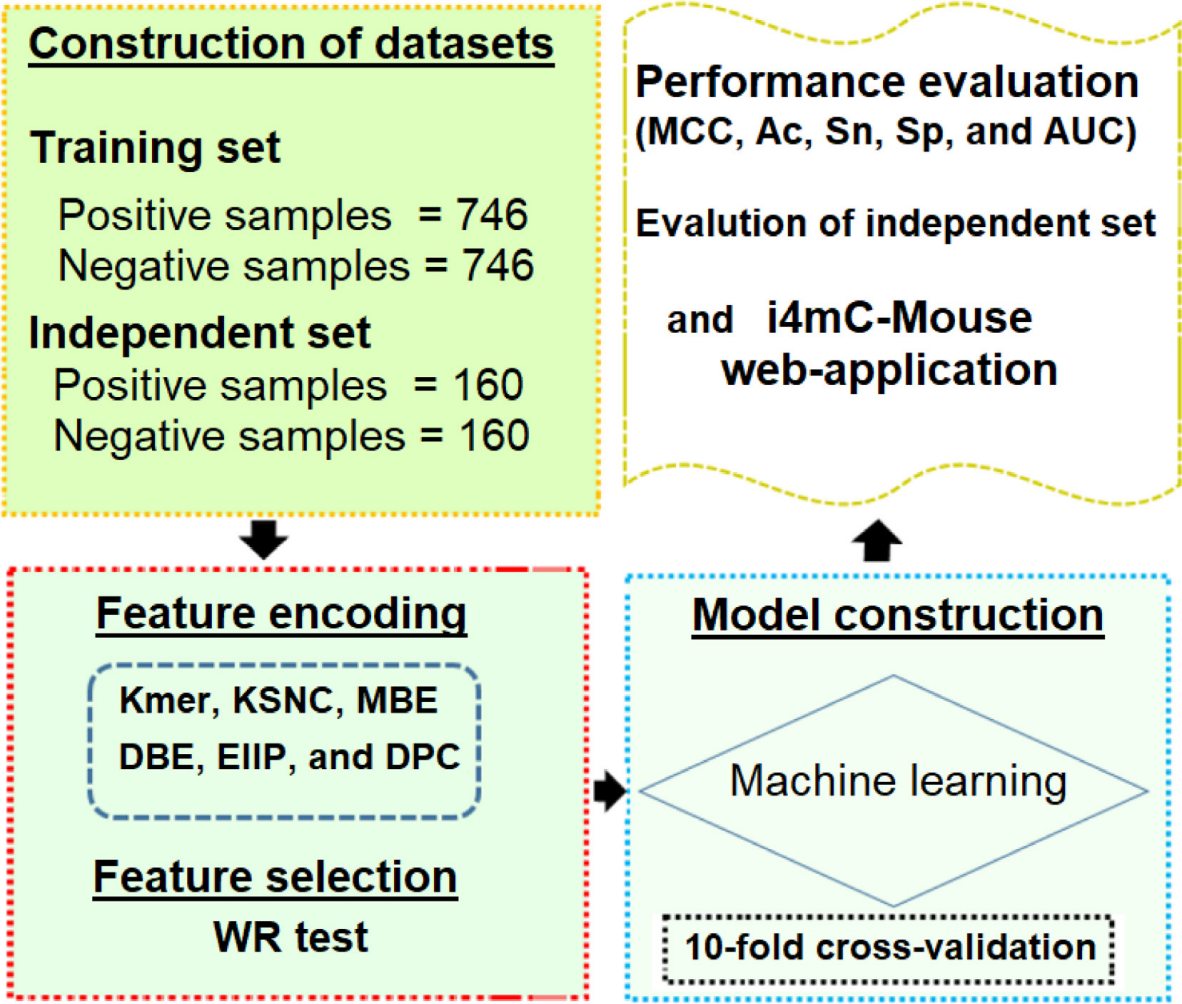
A computational framework of the i4mC-Mouse. It includes three steps: (i) dataset construction; (ii) selection of six different encoding schemes that convert DNA sequences into numerical feature vectors; and (iii) model evaluation and construction using a CV test. Then, construction of a webserver for the final prediction model (i4mC-Mouse).

Motivated by the aforementioned problems, in this work, we have implemented a computational tool called i4mC-Mouse for the identification of 4mCs in the genome of mouse. A workflow of the proposed i4mC-Mouse is summarized in Fig. 1. Initially, six probabilities of 4mC sites were predicted by using a random forest (RF) classifier in conjunction with the k-mer nucleotide (NT) arrangement (Kmer), k-space NT composition (KSNC), NT mono binary encoding (MBE), dinucleotide binary encoding (DBE), electron–ion pseudopotentials (EIIP), and dinucleotide physicochemical composition (DPC). Secondly, to select the successive feature vectors, the Wilcoxon rank sum test (WR) was accessed. Finally, the four (Kmer, KSNC, MBE and EIIP) models evaluated the probability scores of 4mC sites and these scores were linearly combined to develop the i4mC-Mouse. Our results on independent test showed that i4mC-Mouse outperformed the existing predictor 4mCpred-EL. Finally, for the convenience of experimental scientists, our proposed model was implemented as a web application.

## 2 Materials and methods

### 2.1 Dataset construction

To develop a sequence-based predictor of 4mCs, a reliable dataset is necessary. To make a fair comparison, we used the previous dataset [11], which was collected from MethSMRT [13]. The DNA sequence windows are set to 41 base pairs (bp) having “C” at the center. To yield a high-quality dataset, we considered the sequences with a modQV score of ≥20 and excluded the remaining sequences. It is worth mentioning that the previous study applied a CD-HIT of 80% [17] and excluded the sequences that share 80% sequence identity. To develop a more reliable model and avoid an overestimation of prediction model, we applied CD-HIT of 70% and excluded the sequences that showed greater than 70% sequence identity. After such screening procedures, we finally obtained the benchmark dataset containing 906 positive samples, which are 74 samples lower than those of the 4mCPred-EL. A subset of 906 non-4mCs were randomly extracted from the non-4mCs. After obtaining the balanced dataset consisting of 906 4mCs and 906 non-4mCs, we divided them into the training and independent sets, such as 80% samples (746 4mCs and 746 non-4mCs) and 20% samples (160 4mCs and 160 non-4mCs), respectively.

### 2.2 Feature encoding

The next crucial step is to represent a DNA sequence as fixed-length feature vectors [18], [19]. Six encoding methods of Kmer, KSNC, MBE, DBE, EIIP and DPC were used. The potential capability of these encodings employed in many domains has already been mentioned in our previous studies [20], [21].

*Kmer:* This encoding has been extensively used in different prediction tasks [15], [22], [23]. In this study, a DNA sample with *L* length is articulated as *D = d_1_, d_2_, d_3_,… d_L_*, *d_i_* is one of the NTs (A, C, G, T, N). Considering tri-, and tetra -nucleotides, the Kmer scheme generated a 750 (=5^3^ + 5^4^) dimensional (D) feature vector. Here the letter ‘N’ signifies a non-standard nucleotide.

*KSNC:* This encoding signifies the frequency NTs information by using the pair-wise similarity searches [23] and widely used in bioinformatics tasks [24], [25], [26]. The NT (A, C, G, T, N) pairs (*nc_i,_* where *i* = 1, 2,…,25) were encoded and standardized as

(1) $$NTpair=\frac{F(nci)}{w-d-1}$$

where *F*(*nc_i_*) is the sum of *nc_i_* privileged 4mC sites. The *w* and *d* are the sequence length and space length between NTs, respectively. For a range of *dmax* is 0 to 3, the KSNC signifies a 100-D feature vector.

*MBE:* The MBE exactly depicts the NT for the sequence of curated samples at each position, where A, T, G, C, and N are represented by (1,0,0,0,0), (0,1,0,0,0), (0,0,1,0,0), (0,0,0,1,0), and (0,0,0,0,1), respectively. In MBE, for a length of NT sequence, a *w* × 5-D vector was generated.

*DBE:* In the DBE scheme, the possible 16 dinucleotides are encoded as 0/1 (four-dimensional vector) [11]. For instance, AT (0,0,0,1), AA (0,0,0,0), GG (1,1,1,1), and AC (0,0,1,0) are encoded [27], [28]. All N pair dinucleotides are regarded as zero. For a sequence of 4mC or non-4mC with a DBE, a 160 {(*w* − 1) × 4} − D vector was generated.

*EIIP:* To encode the electron–ion energies in the DNA, Nair and Sreedharan developed EIIP [29]. In this study, EIIP values were encoded as follows: A (0.1260), C (0.1340), G (0.0806), T (0.1335), and N (0.0000). The EIIP scheme transformed a sequence into a *w*-D feature vector.

*DPC:* Fifteen types of DPC were collected from the recent publications [20], [21]. The physicochemical properties are encoded as a 375 (25 dinucleotides × 15 physicochemical properties)-D vector.

### 2.3 Feature selection

Inclusion of non-informative and noisy feature might cause unsatisfied prediction performances [30], [31]. In fact, there are several feature selection and ranking approaches, such as Chi-square, mRMR, and WR test. In this work, the WR feature selection method was used [32].

### 2.4 Machine learning classifier

The computational model employed herein was constructed by using the RF algorithm [33]. The RF classifier is widely used in various biological problems [34], [35], [36], [37], [38], [39], [40]. The RF classifier is a collaborative model consisting of many regression and classification trees, and the prediction performances are enhanced by increasing the number of weak CART classifiers. In this study, the RF package ‘randomForest’ (https://cran.r-project.org/) was used.

It is crucial to compare the proposed RF-based models with other commonly used ML-based models, i.e. Naive Bayes (NB) [41], [42], SVM [37], [43], k-nearest neighbor (KNN), and AdaBoost (AB). The NB and AB classifiers were performed in R programming (https://www.r-project.org/), while the KNN classifier was implemented in our house PERL program. The SVM*^light^* was used to build the SVM algorithm [38]. Notably, all these classifiers are extensively applied to various prediction problems [44], [45], [46], [47], [48].

### 2.5 Combined model

To increase the prediction performance of the proposed model, we linearly combined the probability scores of the six, single encoding-based models, as given by:

(2) $$Combined\;\left(s\right)=\sum_{i=1}^{6}w_{i}x_{i}\left(s\right),\;\sum_{i=1}^{6}w_{i}=1$$

where Combined (*s*) specifies the combination of the 6 scores evaluated by the single encoding scheme-employing MLs, *w_i_* characterizes the weight of the *i*-th encoding model and *xi*(s) specifies the ML scores of sample *s* based on the *i*-th encoding model. These weight values were adjusted based on the AUC values via 10-fold cross-validation (CV) tests.

### 2.6 Evaluation metrics

Four statistical metrics: Matthews correlation coefficient (MCC), accuracy (Ac), sensitivity (Sn), and specificity (Sp) were used to evaluate the performance of the predictors as follows [39], [49], [50], [51], [52]:

(3) $$\;\text{MCC}=\frac{\text{n(TP)}\times \text{n(TN)}-\text{n(FP)}\times \text{n(FN)}}{\sqrt{\text{[n(TN)}+\text{n(FN)]}\times \text{[n}\left(\text{TP)}+\text{n(FP}\right)\text{]}\times \text{[n}\left(\text{TN)}+\text{n(FP}\right)\text{]}\times \text{[n(TP)}+\text{n(FN)]}}}$$

(4) $$\text{Ac}=\frac{\text{n(TP)}+\text{n(TN)}}{\text{n(TP)}+\text{(FN)}+\text{n(FP)}+\text{n(TN)}}$$

(5) $$\;\text{Sn}=\frac{\text{n(TP)}}{\text{n(TP)}+\text{n(FN)}}$$

(6) $$\text{Sp}=\frac{\text{n(TN)}}{\text{n(TN)}+\text{n(FP)}}$$

where n(TP) and n(TN) specify the numbers correctly predicted samples of 4mCs and non-4mCs, respectively. n(FP) and n(FN) specify the numbers incorrectly predicted samples of 4mCs and non-4mCs, respectively.

## 3 Results and discussion

### 3.1 Nucleotide preference analysis

**Fig. 2:**
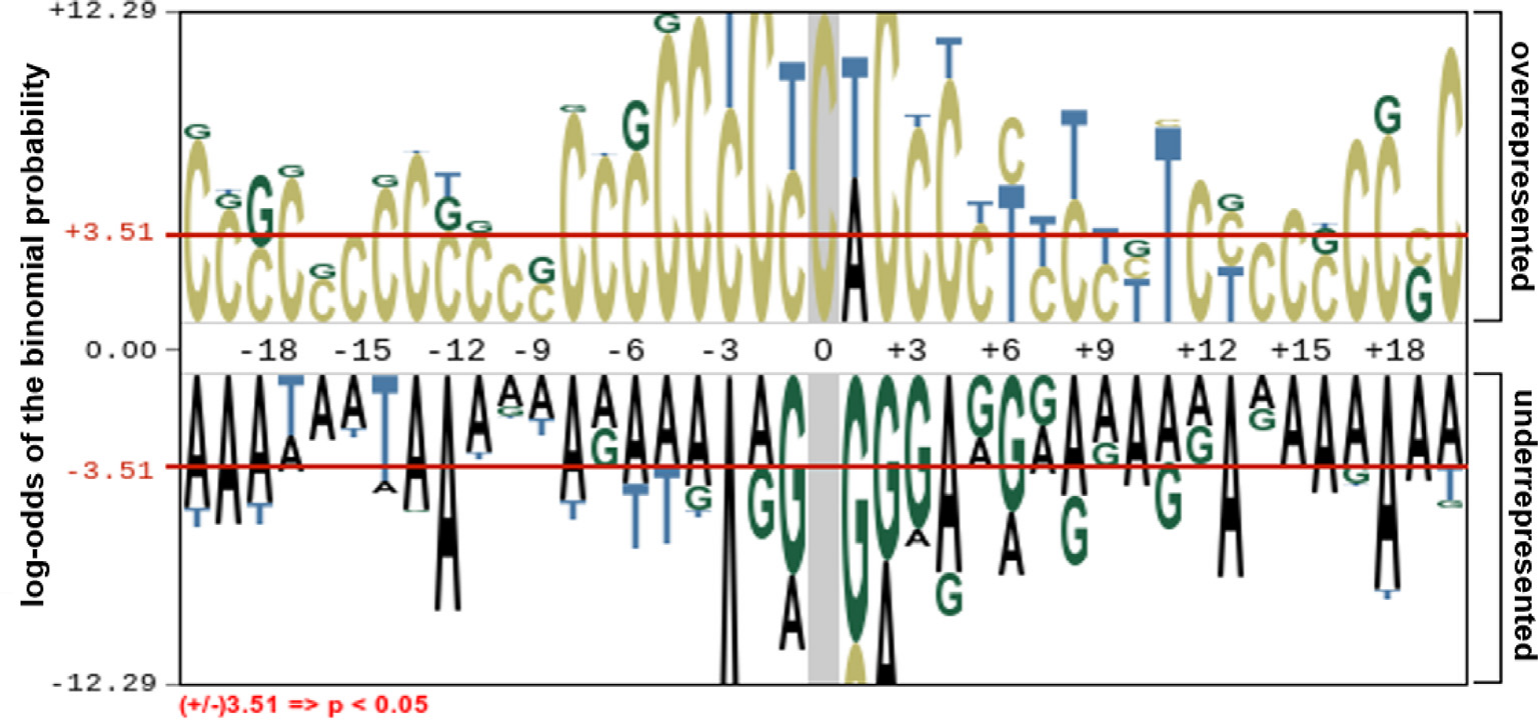
Sequence logo representation of 4mC samples. The 20 upstream and 20 downstream DNA residues surrounding the mouse 4mC site were analyzed.

We aim to develop a computational model for discriminating 4mC samples from non-4mC ones. Therefore, we sought to determine the composition of sequence preferences between the 4mC and non-4mC samples by using the pLogo software [53]. The pLogo examines the statistically significant differences in position-specific NTs (*p* < 0.05). As seen in Fig. 2, the C base was overrepresented compared to the other bases in the 4mC samples and the A base was under-represented compared to the other bases, while the G and T bases were observed at both the over- and underrepresented positions. In summary, the over- and under-represented A and C bases were considerably varied between the 4mC and non-4mC samples, suggesting the importance of position-specific preferences of nucleotide base pairs, which is consistent with the previous study [11].

### 3.2 Performance evaluation of i4mC-Mouse

**Fig. 3:**
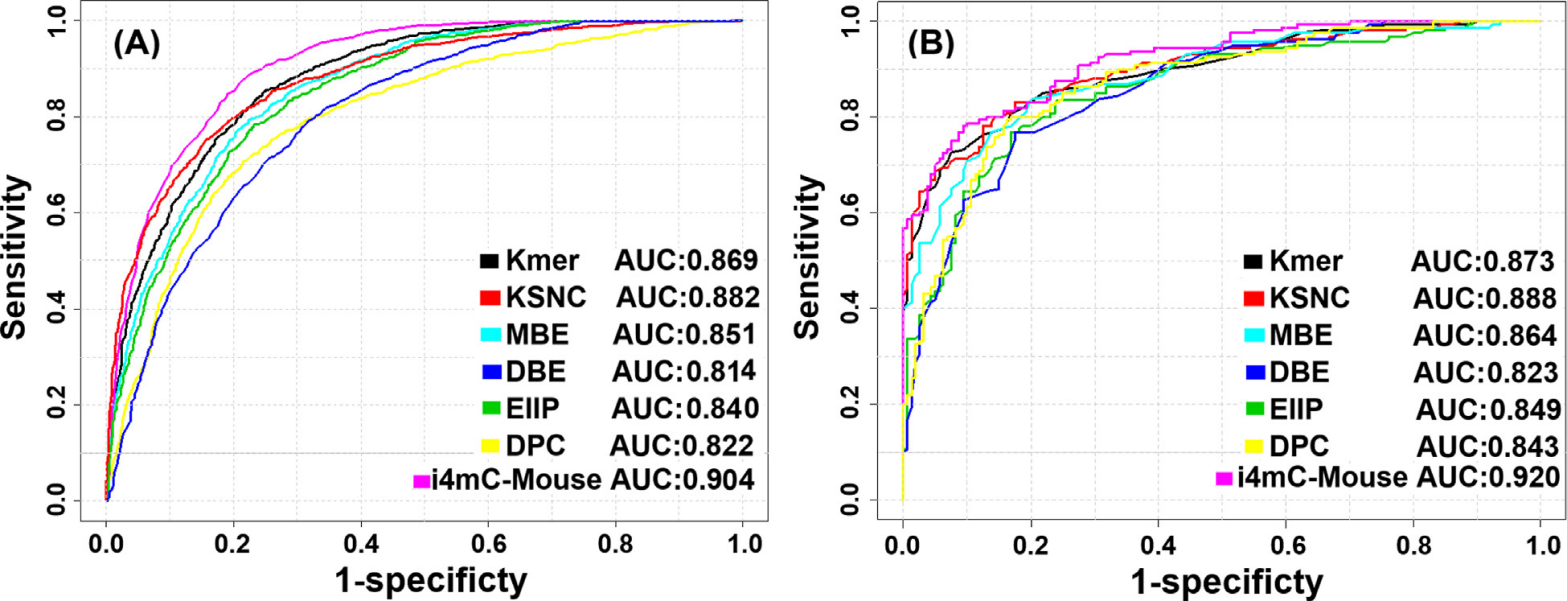
Performance comparisons of single encoding-based models and i4mC-Mouse. The ROC curves were evaluated on the training dataset by a 10-fold CV test (A) and independent dataset (B).

First, the training dataset was converted into feature vectors by using six schemes (Kmer, KSNC, MBE, DBE, EIIP, and DPC) and individually inputted to a RF classifier. Second, we evaluated the successive feature vectors for the six, single encoding models by 10-fold CV tests. To reduce the feature dimension and improve the prediction performance, we carried out the WR test approach to select an optimal feature set on each encoding and compared its performance with the control. As shown in Table S1, the feature selection improved the performance on the three encodings (Kmer (160D), KSNC (80D) and DPC (110D)), while the remaining three encodings (MBE, DBE and EIIP) did not outperform their controls. Therefore, we used three optimal feature set-based models for the subsequent analysis. Fig. 3 and Table 1 show the prediction performances of the six, single encoding-based models and the combined model (i4mC-Mouse). The six, single encoding-based models of Kmer, KSNC, MBE, DBE, EIIP and DPC provided AUCs of 0.869, 0.882, 0.851, 0.814, 0.840 and 0.822, respectively. In terms of Ac and MCC, the KSNC encoding outperformed the other encodings, where the AUC of the KSNC was approximately ~1–7% higher than the AUCs of the other encodings.

**Table 1:** Prediction performances of the i4mC-Mouse model and the single encoding-based RF models.

Methods	MCC	Ac (%)	Sn (%)	Sp (%)	AUC	P-value
Kmer	0.566	74.81	59.53	90.10	0.869	0.011
KSNC	0.602	76.90	63.42	90.30	0.882	0.063
MBE	0.486	71.20	53.81	88.61	0.851	0.006
DBE	0.432	69.13	48.11	90.10	0.814	0.001
EIIP	0.473	70.80	52.31	89.21	0.840	0.001
DPC	0.428	69.21	49.91	88.52	0.822	0.001
i4mC-Mouse	0.651	79.30	68.31	90.20	0.904	–

* i4mC-Mouse specifies the linear arrangement of the RF scores for Kmer, KSNC, MBE, DBE, EIIP, and DPC encodings and their weight values are 0.10, 0.45, 0.25, 0.00, 0.20, and 0.00, respectively.

In the combined model, a linear regression model was used to integrate the six RF probability scores, as mentioned in the method section, where the weight coefficients of the Kmer, KSNC, MBE, DBE, EIIP, and DPC schemes are 0.10, 0.45, 0.25, 0.00, 0.20 and 0.00, respectively. Notably, our approach excluded the two models (DBE and DPC) by assigning weight 0.00 and considered the remaining four models. The contribution of Kmer, KSNC, MBE and EIIP are 10%, 45%, 25%, and 20%, respectively, in the final prediction. As noticed in Table 1, at a Sp control of 90.42%, the i4mC-Mouse yielded MCC, Ac, Sn, and Sp of 0.651, 79.30% 68.31%, and 90.42% respectively. To show the advantage of our approach, we computed the statistically significant differences between the i4mC-Mouse and each single encoding-based model using two-tailed *t*-test [54]. The i4mC-Mouse outperformed the five models at a *p*-value of <0.05, except the KSNC model at a *p*-value of 0.063.

### 3.3 Effect of ML algorithms on prediction performances of the combined model

**Fig. 4:**
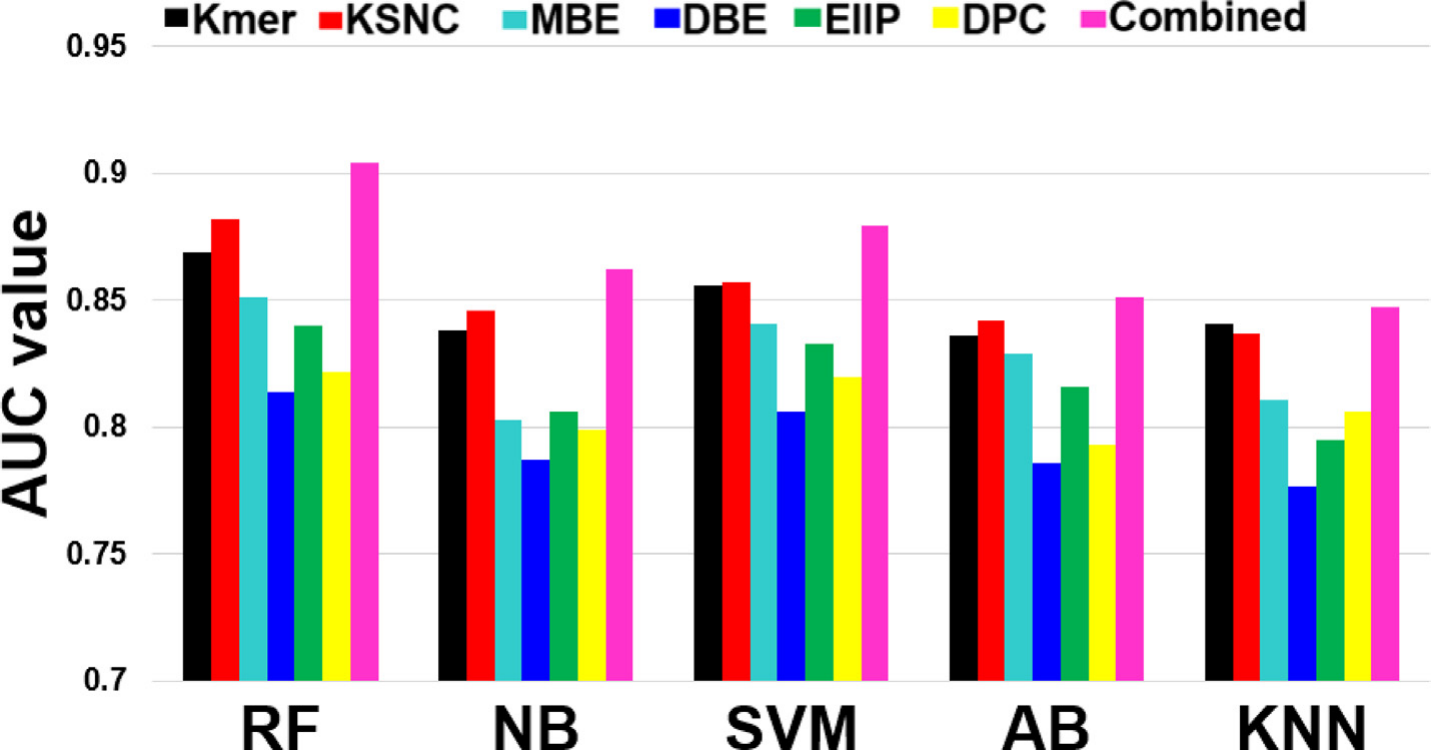
Effect of different ML algorithms on the AUC values of the six single encoding-based models and i4mC-Mouse. The performances were evaluated on the training datasets by a 10-fold CV test.

We applied the above procedure (the construction of six encoding-based models and combined models) to other commonly used four classifiers (NB, SVM, AB and KNN) and compared their performances with the RF-based models. Instead of selecting default ML parameters, 10-fold CV was employed to optimize their respective ML parameters on each encoding-based classifier. Finally, an optimal model was obtained for each classifier, whose performances are shown in Fig. 4. We noted that the combined model for each classifier performed better than the individual encoding-based model, indicating the integration of multiple information is effective in achieving the best performance. Furthermore, comparison among the combined models with five different classifiers showed that the RF achieved the best performance, while the SVM was comparable to the RF model. Specifically, AUCs of the RF (i.e. i4mC-Mouse) were ~1–5% higher than those of any other combined models, demonstrating that the RF model is the most suitable for the i4mC prediction.

### 3.4 Comparison of i4mC-Mouse with 4mCpred-EL on the independent dataset

We compared the proposed i4mC-Mouse with the existing method (4mCpred-EL) on the same independent dataset consisting of 160 4mCs and 160 non-4mCs, as shown in Table 2. We directly submitted to the independent dataset to the 4mCpred-EL web server. The 4mCpred-EL yielded 79.10% Ac, 75.72% Sn, 82.51% Sp, 0.584 MCC, and 0.881 AUC, while the i4mC-Mouse provided 81.61% Ac, 80.71% Sn, 82.52% Sp, 0.633 MCC, and 0.920 AUC. The i4mC-Mouse outperformed the 4mCpred-EL with increased ratios of >3%, >5% and >5% on Ac, Sn and MCC, respectively. The better performance of the i4mC-Mouse would be due to the followings: selection of an appropriate classifier, a linear combination of single encoding-based models, and reduction of dataset redundancy.

**Table 2:** Comparison between the i4mC-Mouse and 4mCpred-EL.

Method	MCC	Ac (%)	Sn (%)	Sp (%)	AUC
4mCpred-EL	0.584	79.10	75.72	82.51	0.881
i4mC-Mouse	0.633	81.61	80.71	82.52	0.920

The performances were evaluated on the independent dataset.

### 3.5 i4mC-Mouse web server

A user-friendly and freely accessible web application was established for the prediction of mouse genome at http://kurata14.bio.kyutech.ac.jp/i4mC-Mouse/. The manuals are as follows: (i) select the exact 41 bp DNA 4mC genome (ii) browse or enter the query sequences from users' own file (FASTA format) to the input page, where a sample is shown our server page, (iii) push the ‘Submit’ button. The server completes the query tasks with the probability scores within one min.

## 4 Conclusions

4mC plays an important role in the DNA modifications and is involved in regulating cell differentiations and gene expression levels. Therefore, accurate identification of 4mC sites is an essential step to understand the exact biological functions. To date, several computational prediction tools have been developed to identify 4mC sites from different species [11], [12], [14], [15], [16], [20], [55], [56], but only one method is available for mouse species. In this study, we have developed a new computational model, called i4mC-Mouse, for improving the prediction of 4mCs in the mouse genome. We employed six encoding schemes of Kmer, KSNC, MBE, DBE, EIIP and DPC to cover various aspects of DNA sequences and optimized the successive features via the WR feature selection method. The final constructed i4mC-Mouse was a linear combination of the predicted probabilities by four, single encoding-based RF-models, where the Kmer, KSNC, MBE and EIIP encodings contributed to 10%, 45%, 25%, and 20%, respectively. On the independent test the i4mC-Mouse outperformed the existing method (4mCpred-EL). The i4mC-Mouse is demonstrated to be the most accurate predictor. Finally, a freely available web application was implemented.

## 5 Author statement

MH and HK conceived the project. MMH and KMS collected and analyzed the datasets. MMH drafted the manuscript. HK, MMH, MB, SW and KMS thoroughly revised the manuscript. All authors approved and read the final manuscript.

## Declaration of Competing Interest

The authors declare that they have no known competing financial interests or personal relationships that could have appeared to influence the work reported in this paper.
