# Supplementary material for: i4mC-Mouse: Improved identification of DNA N4-methylcytosine sites in the mouse genome using multiple encoding schemes
Source: Comput Struct Biotechnol J. 2020 Apr 8;18:906–12. doi: 10.1016/j.csbj.2020.04.001 (PMC7168350; doi:10.1016/j.csbj.2020.04.001)
Supplement: Supplementary data 1 [file mmc1.docx]

**Table S1.** AUCs of i4mC-Mouse without and with features selection.

| Measurement | Without feature selection | With feature selection |
| --- | --- | --- |
| Kmer | 0.849 | 0.869 |
| KSNC | 0.857 | 0.882 |
| MBE | 0.851 | 0.848 |
| DBE | 0.814 | 0.812 |
| EIIP | 0.840 | 0.839 |
| DPC | 0.811 | 0.822 |

By the WR approach, we selected top 160, 80, 110, 90, 70, and 30 informative features from the Kmer, KSNC DPC, MBE, DBE, and EIIP encodings, respectively.
